# No evidence for WU polyomavirus infection in chronic obstructive pulmonary disease

**DOI:** 10.1186/1750-9378-4-12

**Published:** 2009-08-28

**Authors:** Felix C Ringshausen, Marei Heckmann, Benedikt Weissbrich, Florian Neske, Irmgard Borg, Umut Knoop, Juliane Kronsbein, Barbara M Hauptmeier, Gerhard Schultze-Werninghaus, Gernot Rohde

**Affiliations:** 1 Clinical Research Group "Significance of viral infections in chronic respiratory diseases of children and adults", University Hospital Bergmannsheil, Department of Internal Medicine III, Pneumology, Allergology and Sleep Medicine, Ruhr-University Bochum, Germany; 2 Department of Medicine, Spital Bülach, Bülach, Switzerland; 3 Institute of Virology and Immunobiology, University of Würzburg, Germany

## Abstract

Human polyomaviruses are known to cause persistent or latent infections, which are reactivated under immunosuppression. Polyomaviruses have been found to immortalize cell lines and to possess oncogenic properties. Moreover, the recently discovered Merkel cell polyomavirus shows a strong association with human Merkel cell carcinomas. Another novel human polyomavirus, WU polyomavirus (WUPyV), has been identified in respiratory specimens from patients with acute respiratory tract infections (ARTI). WUPyV has been proposed to be a pathogen in ARTI in early life and immunocompromised individuals, but so far its role as a causative agent of respiratory disease remains controversial.

The objective of our study was to determine the prevalence of WUPyV infections in adult hospitalized patients with acute exacerbation of chronic obstructive pulmonary disease (COPD) and to establish its potential clinical relevance by comparison to patients with stable COPD hospitalized for other reasons than acute exacerbation of COPD (AE-COPD).

A total of 378 respiratory specimens, each 189 induced sputum and nasal lavage samples from 189 patients, who had been recruited in a prospective 2:1 ratio case-control set-up between 1999 and 2003, were evaluated for the presence of WUPyV DNA by real-time PCR.

In the present study we could not detect WUPyV DNA in 378 respiratory specimens from 189 adult hospitalized patients with AE-COPD and stable COPD in four consecutive years.

Persistence of viral replication or reactivation of latent WUPyV infection did not occur. WUPyV may not play a major role in adult immunocompetent patients with AE-COPD and stable COPD.

## Findings

Polyomaviruses are small, non-enveloped viruses with a circular double-stranded DNA genome of approximately 5,000 base pairs. They are known to be capable of immortalizing animal and human cell lines. Their oncogenic potential has been demonstrated in vitro and in various animal cancer models and is accomplished by the integration of viral DNA into the host cell genome. Expression of the viral T-antigen is mandatory for cell transformation [1]. In the last years there has been a re-emergence of interest in human polyomaviruses as possible carcinogens as three novel polyomaviruses have been described in humans. While KI and WU polyomavirus have initially been detected in respiratory specimens [2,3], the Merkel cell polyomavirus (MCPyV) was observed to be clonally integrated into Merkel cell carcinomas (MCC), a rare but aggressive human skin cancer of neuroendocrine origin [4]. Meanwhile, MCPyV has also been described in respiratory specimens [5,6], small cell lung cancer tissue [7], and there is increasing evidence for a strong association between MCPyV and MCC [8-12]. Recently a first study reported the detection of KIPyV DNA in lung cancer specimens [13], and many more studies targeting different human polyomaviruses, cancer entities and populations may be anticipated in the near future. However, the detection in the respiratory tract is not a unique feature of KI-, MC- and WUPyV and transmission by the respiratory route has already been suggested for the first two human polyomaviruses, BK and JC virus [14-16]. In 2007, WU polyomavirus (WUPyV) was identified in respiratory specimens from patients with acute respiratory tract infections (ARTI) [2]. It has been proposed to be a relevant pathogen in ARTI in early life and immunocompromised individuals, but so far its role as a causative agent of respiratory disease remains controversial as it was also found in healthy asymptomatic individuals [17,18]. WUPyV infections appear endemic worldwide [2], detection frequencies vary from 0.4% [19] to 7% [20] and coinfections with other respiratory viruses are common [21].

The aim of the present study was to determine the prevalence of WUPyV infections in adult hospitalized patients with acute exacerbation of chronic obstructive pulmonary disease (COPD) and to establish its potential clinical relevance by comparison to patients with stable COPD hospitalized for other reasons than acute exacerbation of COPD (AE-COPD).

A total of 378 respiratory specimens, each 189 induced sputum and nasal lavage samples from 189 patients were retrospectively evaluated for the presence of WUPyV DNA. Subjects with AE-COPD and stable COPD had been recruited in a prospective case-control study in a 2:1 ratio between October 1999 and April 2003. Underlying criteria, definitions and procedures have been the same as described previously [22]. Notably, patients with thoracic malignancies were excluded from the study. The induced sputum and nasal lavage samples were neatly stored in aliquots at -70°C until further processing. DNA was extracted from the samples using the QIAamp DNA Blood Mini Kit (Qiagen, Hilden, Germany) and stored at -20°C for further testing. Although the amplifiability of the assayed sample DNA was recently demonstrated by the detection of another novel respiratory agent, the human bocavirus [23], the human β-globin gene was amplified as a cellular gene on a LightCycler PCR platform (LightCycler^® ^Control Kit DNA, Roche, Mannheim, Germany) in order to demonstrate the integrity and sufficient quality of DNA in the assayed specimens. Primers and probe for the WUPyV real-time PCR assay were selected from the highly conserved C-terminal region of the large T-antigen that has been used for a qualitative WUPyV PCR previously [24]. Blasting of primer and probe sequences against GenBank excluded significant homologies with other organisms. The real-time PCR was performed in a final volume of 25 μl consisting of 5 μl of extracted DNA, the primers WU2958s (CCTGTTAGTGATTTTCACCCATGTA) and WU2865a (TGTCAGCAAATTCAGTAAGGCCTATATAT) at a final concentration of 400 nM, the probe WU2925s-TM (6FAM-AAAGTTGTGTATTGGAAAGAACTGTTAGACA-TAMRA) at a final concentration of 100 nM, and 1× Quantitect probe master mix (Qiagen, Hilden, Germany) as described previously [25]. The cycling conditions were 50 cycles with 30 s at 95°C and 60 s at 60°C after a preheating step of 15 min at 95°C. A plasmid containing the PCR product obtained with the primers AG0048 and AG0049 [2] cloned into the vector pCR2.1-TOPO (Invitrogen, Karlsruhe, Germany) was used as positive control and for the standard curve. Strict laboratory procedures were implemented to prevent PCR contamination. One negative control was amplified for every five samples. Plasmid spiking experiments were conducted in order to exclude PCR inhibition by induced sputum and nasal lavage samples. Data analysis was performed using SPSS, version 11.5 (SPSS Inc., Chicago, Illinois). Categorical data were compared by Pearson's chi-squared or Fisher's exact test, where appropriate. Normal distribution in continuous variables was determined with the Kolmogorov-Smirnov test and differences were subsequently determined either with the student's t-test or the Mann-Whitney-U test. All p values were calculated two-sided with statistical significance set to p < 0.05. The study was approved by the ethics committee of the Ruhr University, Bochum, Germany. All study participants gave their written informed consent prior to study inclusion.

The standard curve was linear over the range from 1+E01 to at least 1+E08 copies per reaction. Spiking experiments using various dilutions of plasmid DNA that were incubated with fresh lower respiratory tract patient samples showed no inhibition of PCR reactions. The lower limit of detection of the applied real-time PCR assay was determined to ten copies per reaction corresponding to 400 copies per mL of starting material. A total of 378 respiratory specimens, each 189 induced sputum and nasal lavage samples, from 189 adult hospitalized COPD patients were investigated for the presence of WUPyV DNA by quantitative real-time PCR. Of those, 123 patients (65.1%) had an acute exacerbation of COPD (AE-COPD) and 66 (34.9%) had stable COPD. The demographic and clinical characteristics of the study population with regard to the COPD status are shown in Table 1. The two groups were comparable in terms of age, sex, body mass index, smoking behavior, pack years and medication. Spirometric data before discharge were available for 77 of 123 patients (62.6%) with AE-COPD, had significantly improved after treatment for acute exacerbation and were comparable to the baseline airflow in the control group. Significant differences were apparent for increased airflow limitation on admission, higher C-reactive protein levels and leukocyte counts in the AE-COPD group (Table 1). WUPyV DNA was not detected in any of the tested samples when using a sensitive real-time PCR assay.

**Table 1:** Demographic and clinical characteristics of the study population

Variables	All		AE-COPD		Stable COPD		P value^a^
	**n**	**%**	**n**	**%**^d^	**n**	**%**^d^	
Patients	189	100	123	65.1	66	34.9	
	**n**	**%**^e^	**n**	**%**^e^	**n**	**%**^e^	
Sex							0.35
Female	39	20.6	28	22.8	11	16.7	
Male	150	79.4	95	77.2	55	83.3	
Smoking behavior							0.34
Active smokers	53	28.0	32	26.0	21	31.8	
Non-smoker	26	13.8	20	16.3	6	9.1	
Ex-smoker	110	58.2	71	57.7	39	59.1	
Oral steroid medication							0.52
Yes	127	67.2	85	69.1	42	63.6	
No	62	32.8	38	30.9	24	36.4	
Inhaled corticosteroids							0.63
Yes	126	66.7	80	65.0	46	69.7	
No	63	33.3	43	35.0	20	30.3	
	**Mean**	**SD**	**Mean**	**SD**	**Mean**	**SD**	
Age (years)	67	10	68	9	65	11	0.17
Body mass index (kg/m^2^)	26.9	5.1	26.8	5.0	27.2	5.2	0.60
	**Median**	**Range**	**Median**	**Range**	**Median**	**Range**	
Pack years^b^	30	2-120	30	2-120	30	2-120	0.71
FEV1_ad _(L)	1.0	0.4-2.6	1.0	0.4-2.2	1.2	0.5 - 2.6	**< 0.0001**
FEV1_ad _(% predicted)	38.0	16.7-79.0	36.7	16.7-79.0	42.9	19.4-77.3	**0.003**
FEV1_dis _(L)	1.2	0.5-2.9	1.2	0.6-2.9	1.2	0.5-2.6	0.53
FEV1_dis _(% predicted)	43.6	18.5-78.9	44.3	18.5-78.9	42.9	19.4-77.3	0.90
CRP (mg/dL)	0.8	0.0-39.8	1.0	0.0-39.8	0.6	0.0-12.9	**0.0002**
Leukocytes/nL	10.5	0.7-27.2	10.9	0.7-27.2	10.1	5.1-24.0	**0.016**
Oral steroid dose (mg)^c^	7.5	0-150	10	0-150	5	0-150	0.098

**Notes: **^a ^P values with statistical significance are printed **bold**. ^b ^Pack years in active and ex-smokers. ^c ^Oral steroid dose in prednisone equivalent before admission. ^d^Percent in line. ^e ^Percent in column. **Abbreviations: **(AE-)COPD = (acute exacerbation of) chronic obstructive pulmonary disease. CRP = C-reactive protein. FEV1_ad _= forced expiratory volume in one second on admission. FEV1_dis _= baseline forced expiratory volume in one second for the control group and before discharge after recovery from exacerbation for the AE-COPD group. SD = standard deviation.

Our findings are in agreement with two recent studies from China [26] and the UK [27], which failed to detect WUPyV DNA in immunocompetent adults. The initial investigation by Gaynor et al. found four adults with altered immune status or multiple comorbidities to be positive for WUPyV [2]. None of the mentioned studies explicitly included patients with AE-COPD or stable COPD. The present population consisted of predominantly elderly COPD patients with severely impaired lung function and concomitant low dose oral steroid medication. In a previous study performed on a comparable population we demonstrated that AE-COPD was significantly associated with the detection of common respiratory viruses, foremost human rhinovirus, influenza virus A and respiratory syncytial virus, and that induced sputum had a higher viral yield than upper respiratory tract specimens in patients with AE-COPD [22]. Though our plasmid spiking experiments showed that PCR reactions were not inhibited by the assayed specimens, the use of diluted plasmid DNA instead of virus titers as a positive control and for the generation of the standard curve is an inevitable limitation of the present study. Infectious WU polyomavirus has yet to be isolated and cell lines susceptible to infection still need to be identified [28]. However, in the present study we could not detect WUPyV DNA in 378 respiratory specimens from 189 adult hospitalized patients with AE-COPD and stable COPD in four consecutive years between 1999 and 2003, whereas recent reports found WUPyV circulating in German, predominantly pediatric populations within and beyond our study period [24,25,29].

Our findings support the hypothesis that primary WUPyV infection is acquired in early life rather than adulthood and suggest that persistence of viral replication or reactivation of latent WUPyV infection is not a common phenomenon in the adult COPD population. Hence, WUPyV may not play a major role in adult immunocompetent patients with AE-COPD and stable COPD. A clear linkage between WUPyV and human disease still remains to be determined.

## Competing interests

The authors declare that they have no competing interests.

## Authors' contributions

FCR conceived and designed the study, performed the statistical analysis, interpreted the data, supervised the study and drafted the manuscript. MH conducted the PCR experiments and revised the manuscript critically for important intellectual content. BW contributed to the study design, helped to establish the PCR assay and revised the manuscript critically for important intellectual content. FN constructed the plasmid for a positive control, contributed to the establishment of the PCR assay and revised the manuscript critically for important intellectual content. IB was involved in the processing of the specimens, UK, JK and BMH recruited the patients and all revised the manuscript critically for important intellectual content. GSW contributed to the study design and supervised the study. GR contributed to the study design, analysis and interpretation of data, supervised the study and revised the manuscript critically for important intellectual content. All authors read and approved the final manuscript.
